# T-lymphocytes from focused ultrasound ablation subsequently mediate cellular antitumor immunity after adoptive cell transfer immunotherapy

**DOI:** 10.3389/fimmu.2023.1155229

**Published:** 2023-07-26

**Authors:** Li-Feng Ran, Xun-Peng Xie, Ji-Zhu Xia, Fang-Lin Xie, Yan-Min Fan, Feng Wu

**Affiliations:** ^1^ Clinical HIFU Center for Tumor Therapy, Second Affiliated Hospital, Chongqing Medical University, Chongqing, China; ^2^ Institute of Ultrasonic Engineering in Medicine, Chongqing Medical University, Chongqing, China; ^3^ Department of Oncology, Nantong Third People’s Hospital, Nantong University, Nantong, Jiangsu, China; ^4^ Nuffield Department of Surgical Sciences, University of Oxford, Oxford, United Kingdom

**Keywords:** high intensity focused ultrasound, ablation, liver cancer, immunomodulation, adoptive cell transfer, immunotherapy, apoptosis, cytotoxic T lymphocyte

## Abstract

**Background:**

Our previous studies found that high-intensity focused ultrasound (HIFU) stimulated tumor-specific T cells in a mouse H_22_ tumor model, and adoptive transfer of the T cells from HIFU-treated mice could subsequently elicit stronger inhibition on the growth and progression of the implanted tumors. The aim of this study was to investigate the mechanism of T cells from focused ultrasound ablation in HIFU-mediated immunomodulation.

**Methods:**

Sixty H_22_ tumor-bearing mice were treated by either HIFU or sham-HIFU, and 30 naïve syngeneic mice served as controls. All mice were euthanized on day 14 after HIFU and splenic T cell suspensions were obtained in each group. Using an adoptive cell transfer model, a total of 1 × 10^6^ T cells from HIFU treated-mice were intravenously injected into each syngeneic H_22_ tumor-bearing mouse twice on day 3 and 4, followed by the sacrifice for immunological assessments at 14 days after the adoptive transfer.

**Results:**

T cells from HIFU-treated mice could significantly enhance the cytotoxicity of CTLs (*p* < 0.001), with a significant increase of TNF-α (*p* < 0.001) and IFN-γ secretion (*p* < 0.001). Compared to control and sham-HIFU groups, the number of Fas ligand^+^ and perforin^+^ tumor-infiltrating lymphocytes (TILs) and apoptotic H_22_ tumor cells were significantly higher (*p* < 0.001) in the HIFU group. There were linear correlations between apoptotic tumor cells and Fas ligand^+^ TILs (r = 0.9145, p < 0.001) and perforin^+^ TILs (r = 0.9619, p < 0.001).

**Conclusion:**

T cells from HIFU-treated mice can subsequently mediate cellular antitumor immunity, which may play an important role in the HIFU-based immunomodulation.

## 1 Introduction

Impaired immunosurveillance is one of the most important factors in the cancer development, and T lymphocyte plays an important role in anti-cancer immunity. As a local ablation approach, high-intensity focused ultrasound (HIFU) ablation has been used in the treatment of cancer patients in the past two decades. It uses ultrasonic energy to raise the temperature between 56°C and 100°C at focus and lead to *in situ* thermal ablation of a targeted tumor. Non-thermal effects can also induce the local tissue destruction in the target due to acoustic cavitation. In addition, large amounts of tumor debris that would contain various tumor antigens are remained *in situ* after HIFU ablation, and gradually reabsorbed as a normal process of healing response. Although it is still unclear whether biological significances may exist during the absorption of the debris, early studies showed that an active immune response to the treated tumor could be subsequently developed, which might help to reduce metastasis and local recurrence after HIFU treatment. HIFU-triggered antitumor immunity has been increasingly investigated in clinical and laboratory settings, and most results have shown that HIFU could boost host antitumor immune response (1–7).

Cytotoxic T lymphocytes (CTLs) are key components of cellular immunity against cancer cells. As the front line of immune defense, the presence of tumor-specific CTLs in both circulating blood and local cancer is a good evidence of host-specific immune response. These CTLs are highly cytotoxic and adoptively transferring them can boost host antitumor immune responses. Our previous studies showed that HIFU could activate tumor-specific T cells in a mouse H_22_ tumor model, and adoptive transfer of the activated T cells elicited stronger inhibition of the growth and progression of the implanted tumors, as well as more survival benefit in the syngeneic tumor-bearing mice (8, 9). During 60-day observation, we found that T cells from HIFU-treated mice could significantly inhibit tumor growth and metastasis rate in the tumor-bearing mice compared with the sham-HIFU and control groups. 60-day survival rate was 86% in the HIFU group, 16% in the control, and 33% in the sham-HIFU. Compared to the sham-HIFU and control groups, the survival rate was significantly higher in the HIFU group. We also found that there was a significant increase in the proportion of both MHC class I tetramer/CD8-positive cells and IFN-γ secreting T cells in HIFU group while comparing with the sham-HIFU and control groups, suggesting that HIFU-based CTLs could present a stronger cell-mediated immune response. However, it is still unknown about the mechanism of the adoptively transferred T cells on the cellular immune status and how they can mediate cancer cell eradication in the tumor-bearing mice. Using an adoptive cell transfer model, this study investigated cellular antitumor immunity in the syngeneic H_22_ tumor mice who had been adoptively transferred with T cells from HIFU-treated mice, and to explore the mechanism that might be involved in HIFU-mediated immunomodulation.

## 2 Materials and methods

### 2.1 Animals and tumor cell line

This animal study was approved by the Experimental Animal Ethnics Committee at the Chongqing Medical University (Chongqing, China), and all experimental procedures adhered to the Animal Welfare Committee guidelines. Female C57BL/6J mice (6–8 weeks old) were provided by the Experimental Animal Center, Chongqing Medical University. They were housed in microisolator cages in a laminar flow unit under ambient light.

A mouse H_22_ hepatocellular carcinoma cell line was obtained from the Institute of Biochemistry and Cell Biology, Chinese Academy of Sciences (Shanghai, China) and cultured in RPMI 1640 medium at 37^°^ C in a humidified 5% CO_2_ atmosphere, supplemented with 10% FCS, 100 U/mL penicillin and streptomycin.

### 2.2 Mouse tumor model and HIFU treatment

An aliquot of 3×10^6^ H_22_ cells were subcutaneously injected into the right flank of each syngeneic C57BL/6 J mouse to establish a mouse H_22_ tumor model. Palpable tumors began to develop on day 3 after the injection and reached the maximum size of 8–9mm in diameter after 7 days.

Sixty tumor-bearing mice were randomly divided into a HIFU group (n=30) and a sham-HIFU group (n=30) on day 7 after H_22_ tumor implantation. Additionally, thirty normal C57BL/6 J mice were used as controls in the study. They were naïve syngeneic mice who didn’t receive either tumor implantation or HIFU treatment. In the HIFU group all mice underwent a complete ultrasound ablation for the subcutaneous tumor. However, in the sham-HIFU group, all mice experienced a sham procedure with no ultrasound irradiation. It was an inactive procedure that was designed to mimic as closely as possible the HIFU procedure, except therapeutic ultrasound was actually turned off during the “treatment”.

Using a handheld transducer, HIFU procedure was carried out at a frequency of 9.5 MHz and acoustic power used for the ablation was 5W. Exposure time ranged from 140 to 180 seconds with median time of 160 seconds.

### 2.3 Collection of T lymphocytes from HIFU-treated mice

60 tumor-bearing mice including 30 in HIFU group and 30 in sham-HIFU, and 30 naïve mice were euthanized at 14 days after HIFU procedure. Peripheral blood was immediately collected, and the spleens were respectively harvested in each group for generating single cell suspensions through a metallic mesh. Using lymphocyte density gradient centrifugation and RBC lysis buffer, the lymphocytic population was then collected, extracted and purified after removing erythrocytes and macrophages. Finally, T cells were isolated by immunomagnetic beads (MagniSort™ Mouse T cell Enrichment Kit, Invitrogen, Carlsbad, CA) in each group, according to the manufacturer’s instruction.

### 2.4 Adoptive immunotherapy with T lymphocytes from HIFU-treated mice

Sixty syngeneic H_22_ tumor mice were used for assessment of the adoptive immunotherapy. They were divided into three groups (HIFU, sham-HIFU and control) and each group had 20 mice. They received the adoptive transfer of the T cell suspensions obtained from the HIFU, sham-HIFU and naïve mice respectively. In brief, a total of 1 × 10^6^ T cells (2 × 10^7^/mL, 0.05 mL) were intravenously injected each time via a tail vein for each mouse, and this adoptive cell transfer was carried out twice on day 3 and 4 after H_22_ tumor implantation. All mice in each group received the same quality and quantity of T-cell suspension from HIFU-treated mice.

### 2.5 Sample collection

All tumor-bearing mice were euthanized on the fourteenth day after adoptive cell transfer. Peripheral blood was firstly collected for assessment of T cell and subsets in each group. The spleen was then harvested, and single-cell suspensions of the spleen were respectively produced by passage through a metallic mesh, followed by removal of erythrocytes and macrophages. Using lymphocyte density gradient centrifugation, the lymphocytic population was collected, extracted and purified in each group for further functional measurements of cytotoxic T-lymphocytes (CTLs). Finally, the implanted tumors were simultaneously harvested, and the viable tumor tissues were dissected using autoclaved surgical instruments. They were washed with 4^°^ C Hank’s balanced salt solution, and then cut into small pieces less than 1 mm^3^. They were mechanically disaggregated with a scalpel and syringe and filtrated through a 200-gauge steel mesh to release single cells. The single-cell suspensions were then prepared in each group for further measurements of activated tumor-infiltrating T cells (TILs) and apoptosis in H_22_ cancer cells.

### 2.6 CTL cytotoxicity assay

Cytotoxicity assay was carried out in the T cells isolated from the spleen of the tumor-bearing mouse after adoptive transfer. They served as effector cells and were incubated with H_22_ cells treated by mitomycin-C as targeted cells. Both cells were co-cultured in 96-well plates for 24 hours at a 20:1 viable effector to target cell ratio. MTT (3-(4,5-dimethylthiazol-2-yl)-2,5-diphenyltetrazolium bromide) colorimetric assay was respectively performed to determine *in vitro* cytotoxicity of CTLs against tumor cells in the three groups. H_22_ cells were served as a positive control, T cells as a negative control and RPMI 1640 medium as a blank control. Each assay was carried out in triplicate.

### 2.7 Enzyme-linked immunosorbent assay (ELISA)

Culture supernatants were also harvested to determine the cytokine release of the CTLs after 24-h incubation of both effector and target cells in each group. Necrosis factor-alpha (TNF-α) and interferon-gamma (IFN-γ) murine ELISA kits were obtained from BD Biosciences (San Jose, CA). According to the manufacturer’s instruction, production of TNF-α and IFN-γ was measured in the supernatants, and each assay was performed in triplicate in each group.

### 2.8 Flow cytometric analysis

Fluorescent antimouse CD3, CD4 and CD8 McAb, FITC-conjugated antimouse Fas ligand (FasL) and Perforin (Pf) McAb were obtained from eBioscience (San Diego, CA). Using EPICS XL flow cytometer (Beckman Coulter, Fullerton, CA), flow cytometry was performed in each group. After linear light scatter signals served to establish the gate on the lymphocyte, a minimum of 10,000 cells of interest from each stained sample were measured for staining positivity. The data were analyzed using Beckman Coulter Expo 32 software.

### 2.9 TUNEL assay

According to the manufacturer’s instruction, terminal deoxynucleotydil transferase dUTP nick and labeling (TUNEL) assay was performed in the H_22_ tumor cells with an apoptosis detection kit (EMD Biosciences, La Jolla, CA). The index of apoptosis was assessed by Beckman Coulter EPICS XL flow cytometer and the ratio of apoptotic cells was determined by comparing the number of positively stained cells with the total number of H_22_ tumor cells.

### 2.10 Statistical analysis

All statistical analyses were performed using the SAS statistical software package version 9.0 (SAS Institute, Cary, NC) and results were presented as means ± standard deviation (X ± SD). Statistical analysis was performed by one -way analysis of variance (ANOVA), and comparisons among the three groups were made by Bonferroni’s multiple-comparison *t*-test as appropriate. Linear regression analysis (Pearson’s coefficient) was used to analyze the correlations between the FasL^+^, Pf^+^ TILs and apoptotic H_22_ cells. Compared to the control group, differences were considered statistically significant when *P* value was less than 0.05 (*p* < 0.05).

## 3 Results

### 3.1 T cells from HIFU-treated mice increase peripheral blood CD4^+^ level and CD4^+^/CD8^+^ ratio

**Figure 1:**
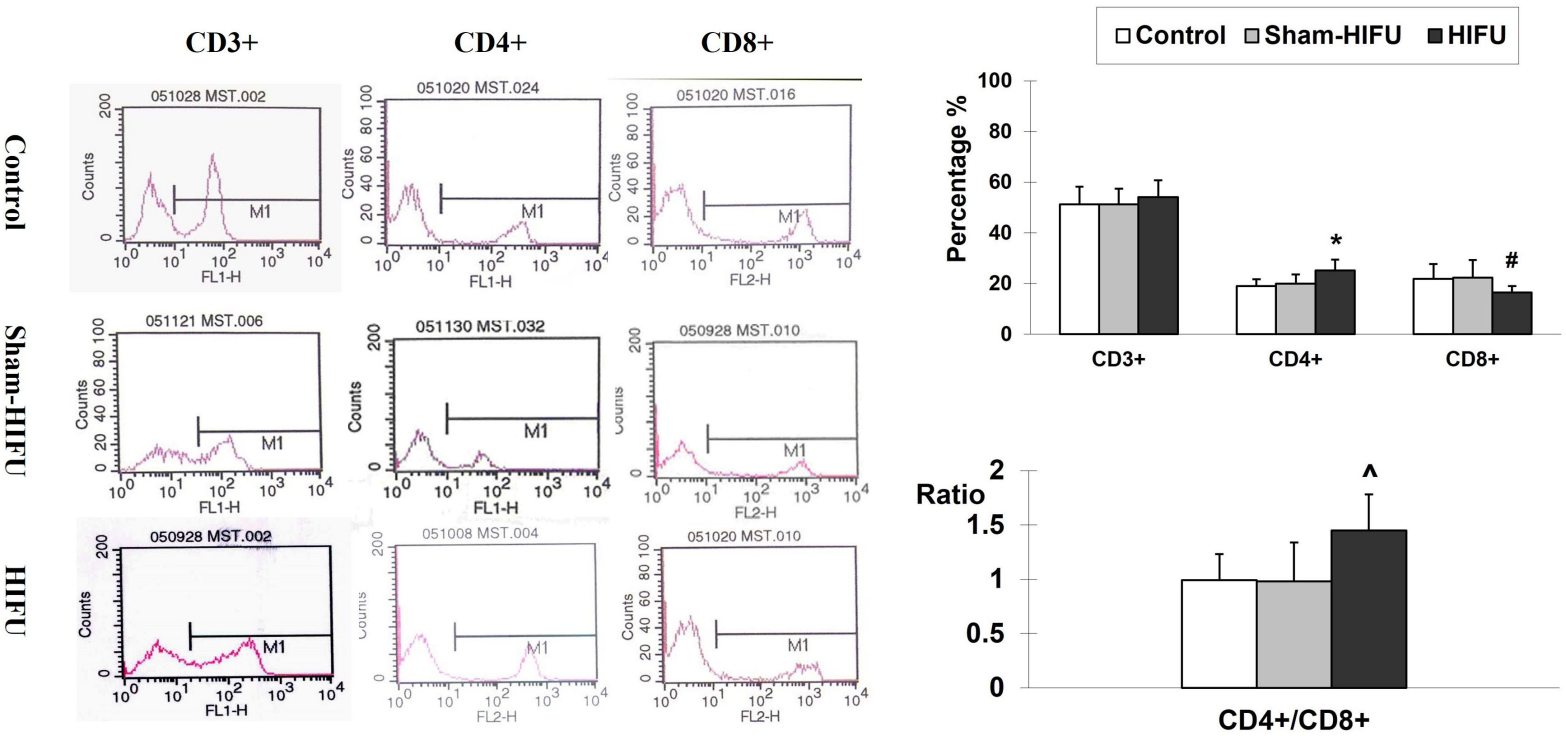
Representative flow cytometry histograms of CD3^+^, CD4^+^ and CD8^+^ in the peripheral blood collected from the control group, sham-HIFU group, and HIFU group after adoptive transfer of T cells from HIFU ablation. T cells from HIFU-treated mice increase peripheral blood CD4^+^ level and CD4^+^/CD8^+^ ratio, as well as decreases CD8^+^ level in the syngeneic tumor-bearing after adoptive cell transfer. In comparison with the control group and sham-HIFU group, * *p* < 0.001; ^ *p* < 0.01; # *p* < 0.05.

The proportion of T cell and subsets was assessed in the peripheral blood of the tumor-bearing mice who had previously received adoptive cell transfer with T cells from HIFU-treated mice. The result was expressed as a percentage of the total white blood cells in the blood sample. As shown in **Figure 1**, T cells from HIFU-treated mice changed the proportion of T cell subset in the peripheral blood. There was significant increases of CD4^+^ level (p < 0.001) and CD4^+^/CD8^+^ ratio (p < 0.01), as well as a significant decrease of CD8^+^ level (p < 0.05) in the HIFU group while compared with the values in the control and sham-HIFU groups. These results suggested that T cells from HIFU-treated mice could increase CD4^+^/CD8^+^ ratio in peripheral blood and present a strong immune response in the tumor-bearing mice.

### 3.2 T cells from HIFU-treated mice increase cytotoxicity and cytokine release of T cells

**Figure 2:**
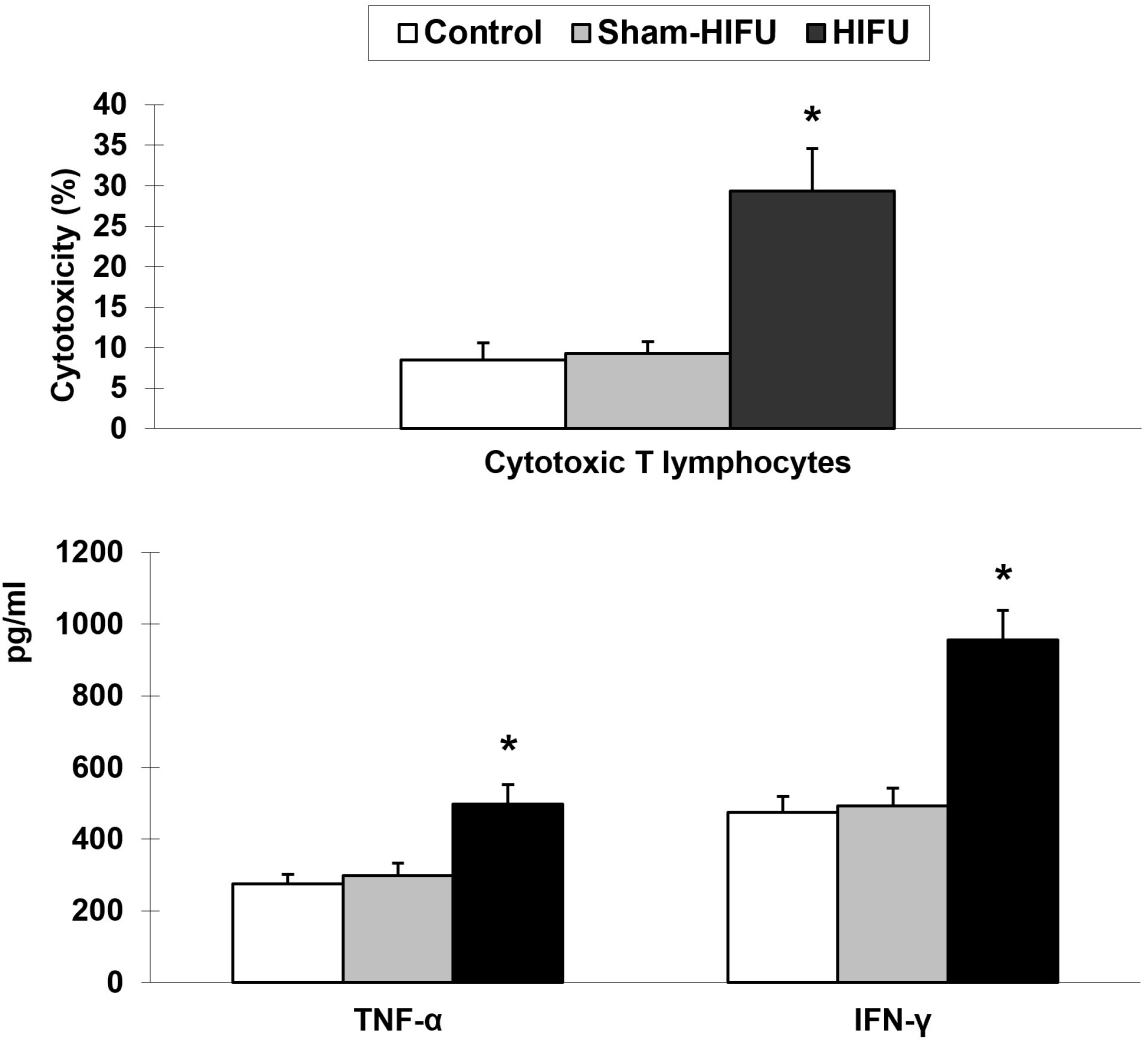
T cells from HIFU-treated mice increase the cytotoxicity and cytokine secretion of cytotoxic T lymphocytes (CTLs) in the tumor-bearing mice after adoptive cell transfer. * *p* < 0.001 in comparison with the sham-HIFU and control groups.

To determine whether T cells from HIFU-treated mice could enhance specific cellular immunity in the syngeneic tumor-bearing mice after adoptive cell transfer, the cytotoxicity of the splenic T cells was evaluated with MTT assay. As shown in **Figure 2**, there was a significant increase of the CTLs toxicity against H_22_ tumor cells (*p* < 0.001) in the HIFU group while compared to the control and sham-HIFU groups. Furthermore, the levels of both TNF-α and IFN-γ released by the CTLs in the culture supernatants, where T cells were incubated with H_22_ cells, were significantly higher (*p* < 0.001) in the HIFU group than those in the control and sham-HIFU groups.

### 3.3 T cells from HIFU-treated mice increase the frequency of FasL^+^ and Pf^+^ TILs

**Figure 3:**
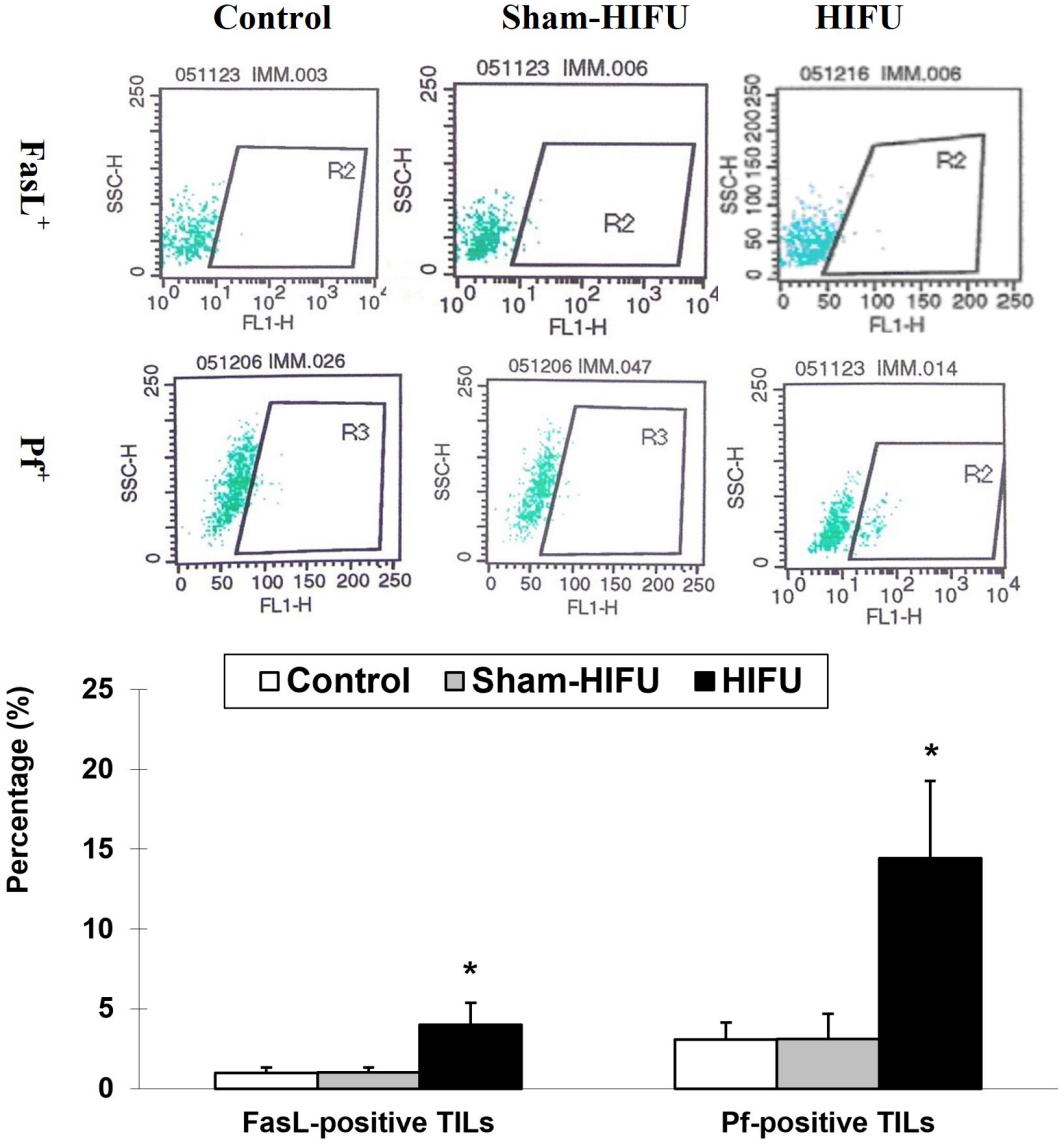
Representative flow cytometry histograms of FaL^+^ tumor-infiltrating T lymphocytes (TILs) and PF^+^ TILs in the control group, sham-HIFU group, and HIFU group after adoptive transfer of T cells from HIFU ablation. T cells from HIFU treated mice increase the frequency of FasL^+^ TILs and Pf^+^ TILs in the tumor-bearing mice after adoptive cell transfer. * *p* < 0.001 in comparison with the control and sham-HIFU groups.

The expression of FasL and Pf on tumor-infiltrating lymphocytes was evaluated to determine the frequency of FasL^+^ TILs and Pf^+^ TILs in the syngeneic tumor-bearing mice after adoptive transfer with T cells from HIFU-treated mice. As shown in **Figure 3**, the number of both FasL^+^ TILs and Pf^+^ TILs were significantly higher (*p* < 0.001) in the HIFU group than those in the control and sham-HIFU groups, indicating that T cells from HIFU-treated mice could significantly infiltrate the implanted tumors, and elicit local cellular antitumor immunity.

### 3.4 T cells from HIFU-treated mice increases apoptosis in H_22_ tumor cells

**Figure 4:**
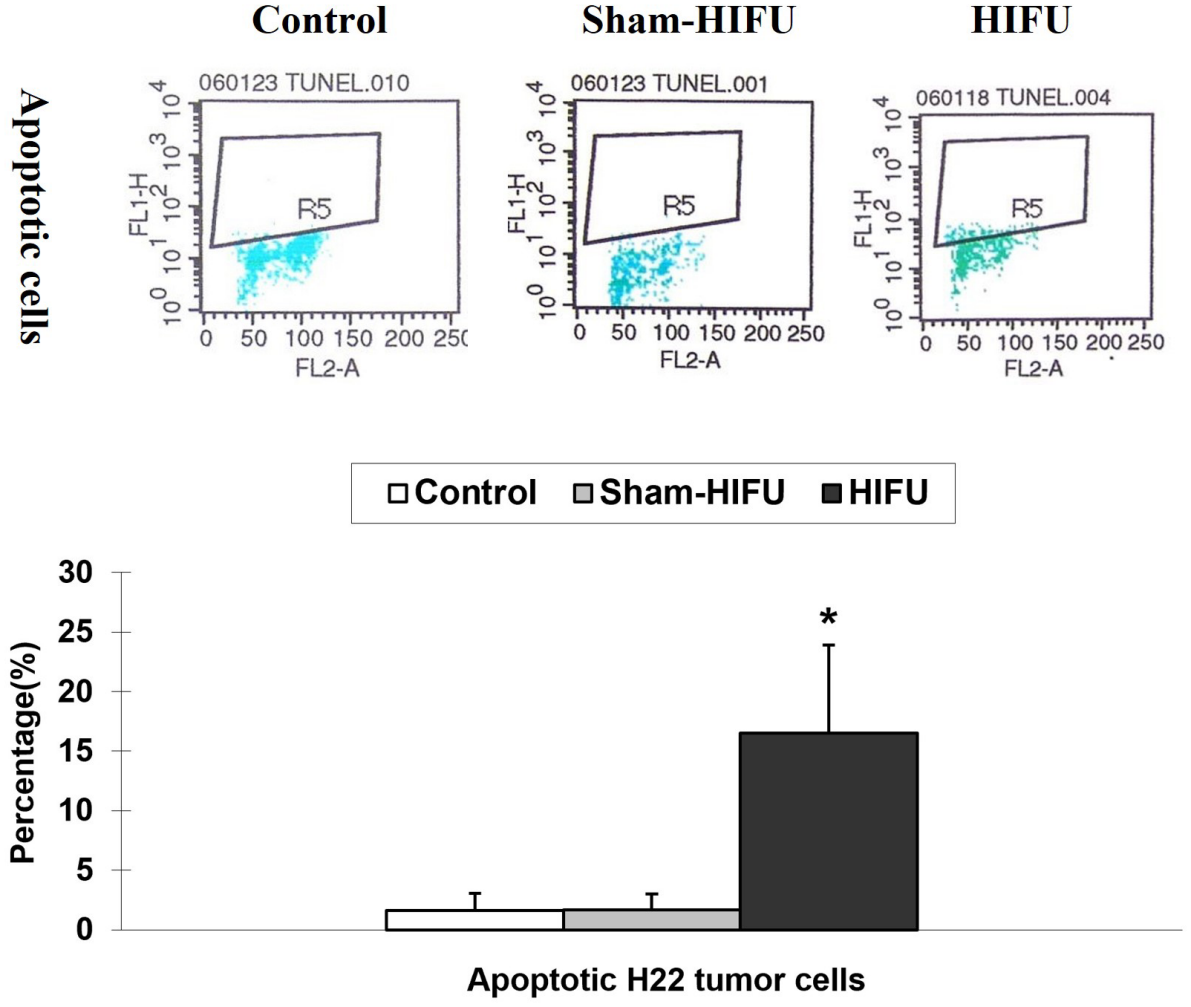
Representative flow cytometry histograms of apoptosis assay in the control group, sham-HIFU group, and HIFU group after adoptive transfer of T cells from HIFU ablation. T cells from HIFU-treated mice increase the number of apoptotic H_22_ tumor cells in the tumor-bearing mice after adoptive cell transfer. * *p* < 0.001 in comparison with the control and sham-HIFU groups.

The number of apoptotic H_22_ tumor cells was evaluated by TUNEL assay in the syngeneic tumor-bearing mice after adoptive transfer with T cells from HIFU-treated mice. As shown in **Figure 4**, the percentage of the apoptotic tumor cells was significantly higher (*p* < 0.001) in the HIFU group than those in the control and sham-HIFU groups, and there was a statistical difference among the three groups. This result revealed that T cells from HIFU-treated mice could significantly induce apoptosis in the implanted tumors after adoptive cell transfer.

### 3.5 Correlations between FasL^+^, Pf^+^ TILs and apoptotic H_22_ tumor cells

**Figure 5:**
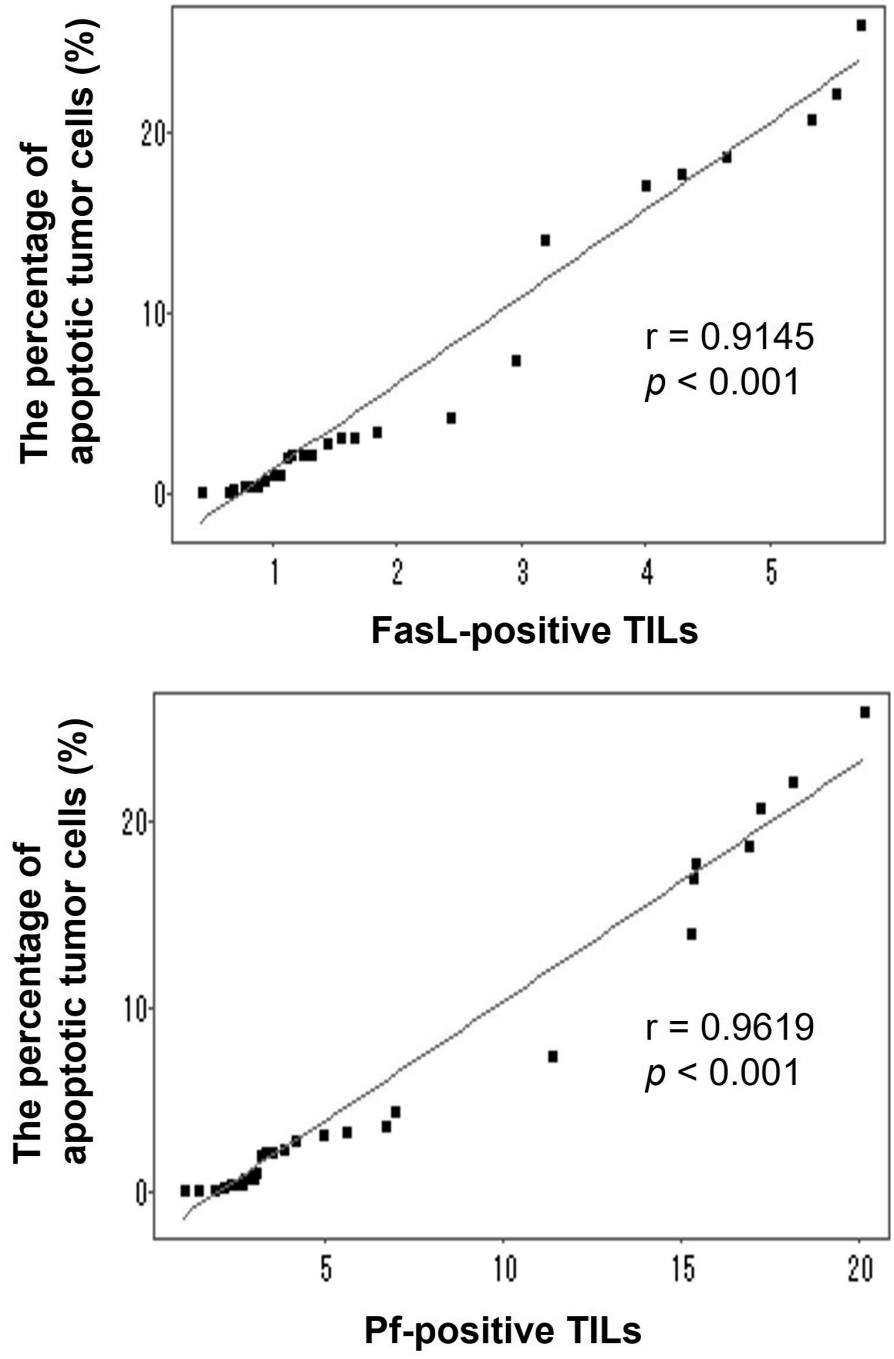
Correlations between the percentage of apoptotic H_22_ tumor cells and the number of either FasL-positive TILs and Pf-positive TILs in the tumor-bearing mice after adoptive transfer with T cells from HIFU-treated mice. Linear correlations were observed between the percentage of apoptotic tumor cells and the number of FasL-positive TILs (r=0.9145, *p <*0.001) and Pf-positive TILs (r=0.9619, *p <*0.001).

To further investigate whether both FasL^+^ TILs and Pf^+^ TILs could contribute to the increased apoptosis of tumor cells in the implanted tumors, we performed the Pearson’s correlation coefficient to analyze the linear regression correlation between the FasL^+^ TILs or Pf^+^ TILs and tumor apoptosis. As shown in **Figure 5**, positive correlations were observed between the percentage of apoptotic H_22_ tumor cells and the number of FasL^+^ TILs (r = 0.9145, p < 0.001), and Pf^+^ TILs (r = 0.9619, p < 0.001). These results suggested that T cells from HIFU-treated mice could directly induce tumor cell apoptosis through both FasL^+^- and Pf^+^-mediated pathways, and subsequently enhance the local cellular immune activities in the tumor-bearing mice.

## 4 Discussion

HIFU is a local intervention approach with aim of intention to ablate a targeted tumor noninvasively. It has been increasingly performed in the clinical management of patients with solid tumors. In addition, HIFU involves a distinctive effect on immune activities, and recent studies have shown that HIFU could trigger host antitumor immunity after local ablation. Although the mechanism is still unclear, it has become more evident that T cells from HIFU-treated mice may play an important role in HIFU-based immunomodulation (10–14). In our previous study, we discovered that after adoptive transfer, T cells from HIFU-treated mice could significantly inhibit tumor growth and increase survival time in the syngeneic tumor-bearing mice (9). Using an adoptive cell transfer model, this study revealed that there were positive correlations between apoptotic H_22_ tumor cells and FasL^+^ TILs (r = 0.9145, p < 0.001), and Pf^+^ TILs (r = 0.9619, p < 0.001), suggesting that T cells from HIFU-treated mice could trigger cellular antitumor immunity and induce apoptosis of the tumor cells through both FasL^+^- and Pf^+^-mediated pathways. These findings will improve understanding and interpreting the mechanism of HIFU immunomodulation.

A good animal model can provide a preclinical tumor microenvironment to address biological interactions between host immune cell and tumor cell. Adoptive cell transfer therapy is a form of cancer immunotherapy that uses autologous immune cells such as T cell (15, 16) and natural killer cell (17, 18) to mediate efficient tumor regression. It consists of T-cell harvesting from tumor or peripheral blood, expanding in an *ex vivo* culture environment and infusing back into the autologous host. In the current study, we used an adoptive T cell transfer model to investigate the mechanism of HIFU-mediated antitumor immunity in the syngeneic tumor-bearing mice. This model involved the administration of the T cells from HIFU ablation in the syngeneic tumor-bearing mice and would serve as a better mirror that was highly relevant to the key mechanism of HIFU-based immunomodulation.

As a crucial mediator of cellular immunity, CTL is a major cytotoxic effector cell in the immune system. The process that CTL uses to attack cancer cells involves releasing death-inducing effector molecules such as FasL granzyme B (GzB) and Pf, and then triggering typical apoptosis of tumor cells through both FasL^+^- and GzB^+^/Pf^+^-mediated pathways (19–22). In this study we found a significantly increased proportion of TILs with FasL^+^ and Pf^+^ expressions (**Figure 3**), as well as apoptotic H_22_ tumor cells (**Figure 4**) in the syngeneic tumor-bearing mice after adoptive transfer of T cells from HIFU ablation. There were positive correlations between the percentage of the apoptotic tumor cells and the proportion of FasL^+^ TILs and Pf^+^ TILs (**Figure 5**), indicating that T cells could directly trigger apoptosis of tumor cells and subsequently drive the cellular antitumor immunity after HIFU treatment. However, as HIFU-based immune response seems to be dominated by the cellular arm of the immune system, other lymphoid and myeloid effector cells including natural killer cells, monocytes and macrophages may be also involved in the induction of antitumor immunity after HIFU ablation. Therefore, further studies are needed to evaluate the role of these cells in HIFU-based immunomodulation.

Our findings may have an important implication in the combination of HIFU with adoptive immunotherapy. In the past two decades, adoptive T cell therapy, which is based on the adoptive transfer of naturally occurring or gene-engineered T cells, has significantly changed the landscape of cancer therapy (23, 24). Engineered T cell receptor therapy and chimeric antigen receptor T cell therapy have increasingly used in the treatment of patients with hematological cancers, and clinical results show that they can induce prolonged tumor eradication (25, 26). Our studies revealed that HIFU could activate T cells, and adoptive transfer of the T cells from HIFU-treated mice could significantly induce tumor apoptosis in the syngeneic tumor-bearing mice. These data provide the fundamental basis for facilitating the development of a novel cell-based immunotherapy in which HIFU, as a local intervention, would be combined with post-HIFU adoptive cell transfer immunotherapy for advanced cancer patients. In this new therapeutic approach, the T cells from HIFU Ablation, which have already existed in HIFU-treated patients, can be collected from peripheral blood, expanded *in vitro* in large numbers, and then reinfused back into the autologous patients.

## 5 Conclusions

In summary, using an adoptive cell transfer model, this study reveals that HIFU may trigger host cellular antitumor immunity, and T cells from HIFU ablation plays an important role in HIFU-based immunomodulation. FasL^+^ and Pf^+^ CTLs can infiltrate the local tumor and induce apoptosis of the tumor cells through both FasL^+^- and Pf^+^-mediated pathways in the syngeneic tumor-bearing mice. Our findings support for developing a novel cell-based immunotherapy in which HIFU, as a local intervention, will be combined with post-HIFU adoptive T cell transfer therapy for patients with advanced-stage cancer.

## Data availability statement

The original contributions presented in the study are included in the article/supplementary materials. Further inquiries can be directed to the corresponding author.

## Ethics statement

The animal study was reviewed and approved by Experimental Animal Ethnics Committee, Chongqing Medical University.

## Author contributions

Conceptualization, L-FR, X-PX, and FW; methodology, L-FR, X-PX, J-ZX, F-LX, and Y-MF; formal analysis, L-FR and X-PX; investigation, L-FR, X-PX, J-ZX, F-LX, and Y-MF; animal samples/resources, L-FR, X-PX, and Y-MF; data curation, L-FR and X-PX; writing—original draft preparation, L-FR and X-PX; writing—review and editing, FW; supervision FW; funding acquisition, FW. All authors contributed to the article and approved the submitted version.
